# Gene-Diet Interactions on Colorectal Cancer Risk

**DOI:** 10.1007/s13668-012-0023-1

**Published:** 2012-07-10

**Authors:** Li-Shu Wang, Chieh-Ti Kuo, Yi-Wen Huang, Gary D. Stoner, John F. Lechner

**Affiliations:** Division of Hematology and Oncology, Department of Medicine, Medical College of Wisconsin, TBRC, Room C4930 8701 Watertown Plank Road, Milwaukee, WI 53226 USA

**Keywords:** Gene-diet interaction, Colon cancer, Colorectal cancer, NADPH oxidases, H_2_O_2_, NOS2, Inflammation, Free radicals, RONS, Epigenome, miRNA, NF-κB, COX-2, Nrf2, WNT, Chemoprevention, Fusobacterium nucleatum bacteria, PTEN, AKT, Macrophages, Neutrophils, K-ras

## Abstract

There has been increasing interest lately in understanding how natural dietary antioxidants affect chemoprevention, and recently, there has been a merging of information about antioxidants, endogenous and exogenous reactive oxygen and nitrogen species (RONS), and inflammation. RONS normally serve the cells as second messengers to regulate many of the intracellular signaling cascades that govern multiple cellular activities. However, when the amount of RONS exceeds the cell’s ability to metabolize/eliminate them, the cell becomes stressed and acquires genetic and epigenetic aberrations and dysregulated intracellular signaling cascades. In addition, there has been a better understanding of the role of tissue inflammation in the carcinogenesis process. Herein we integrate these fields to explain where RONS arise and how natural dietary antioxidants are principally working through refurbishing pathways that use RONS as second messengers.

## Introduction

Reactive oxygen (ROS) and nitrogen (NOS) species (RONS) normally regulate many of the intracellular signaling cascades that govern multiple cellular activities. However, when the amount of RONS exceeds the cell’s ability to metabolize/eliminate them, the cell becomes stressed and acquires genetic and epigenetic aberrations. Excess RONS also dysregulated intracellular signaling cascades. Thus, antioxidation of excess levels of RONS is crucial for reducing the risk of developing cancer. The primary rationale for this conclusion is the marked increased risk of developing cancer in patients with ulcerative colitis and Crohn’s disease [1, 2••]. A strong case has also been made for a major role of inflammation and RONS in the carcinogenesis of the more prevalent sporadic form of colon cancer [3]. Specifically, McLean and associates [4•] recently reported an extensive immunohistochemical study that clearly showed significant infiltration of proinflammatory cells, including macrophages expressing inducible nitric oxide synthase (NOS2; also known as iNOS) in adenomatous lesions. NOS2 is an enzyme that converts arginine to citrulline plus the radical nitric oxide (NO^●−^). The radical itself is oxidative, and also because it generates the radical peroxynitrite (OONO^−^) when it combines with O2^●−^.

Other support for a role of inflammation and RONS in colon carcinogenesis is that NSAIDs reduce the risk of developing this disease [5], and the frequently noted overexpression of NADPH oxidase enzymes (NOX) that produce superoxide (${\text{O}}_2^{ \bullet - }$) and H_2_O_2_, which dissociates to the HO^−^ radical [6]. Thus, inflammatory-associated RONS are a key player in the genesis of this disease. Chemopreventative foods and their active constituents have been shown to decrease the number and size of colon cancer in animal models and recently in humans as well. The paramount feature of the vast majority of these chemopreventive substances is their ability to antagonize proinflammatory cytokines and chemokines and reduce the level of RONS. Therefore, it is expected that molecular pathways most commonly affected by chemopreventive agents would be those that are dysregulated by proinflammatory factors and free radicals.

## The Inflammatory Microenvironment

The primary condition that initiates stress to epithelial cells is an inflammatory microenvironment [1, 2••, 3, 4•, 7–14], wherein infiltrating lymphocytes and macrophages raise the level of RONS to amounts that exceed the ability of the cell to eliminate them by its antioxidant phase II enzymes (eg, superoxide dismutase, catalase, glutathione peroxidase, and peroxiredoxins, glutathione-S-transferase, quinone oxidoreductase, epoxide hydrolase, and others). The inflammatory microenvironment of colon lesions contains many RONS-producing cell types, including neutrophils, monocytes, T and B lymphocytes, macrophages, and natural killer cells. Some of these cells release a number of growth factors and cytokines (eg, transforming growth factor-β [TGF-β]), and chemokines (eg, CCL2, CCL7, CCL3, CCL4, CXCL12, and interleukin [IL]-8), along with tumor necrosis factor (TNF)-α. These factors chemoattract monocytes and macrophages. When chronically activated, this inflammatory/oxidative environment becomes a relentless cycle that results in genetic and pathological damage to the apposition epithelium [14].

Some of the procarcinogenic activity of the infiltrating lymphocytes is caused by their release of ROS as they phagocytize *Fusobacterium nucleatum* bacteria. Previously known as a periodontal pathogen, this microbe is a rare member of the fecal microbiota of noncancerous patients. However, it is prevalent in polyps and is a major resident in tumor tissue. When tested with peripheral blood lymphocytes, the oxidative burst produced by phagocytizing *F. nucleatum* was significantly greater than those from other species of bacteria. At the same time, this microbe indirectly caused differential expression of 208 genes in the lymphocyte. Seven percent of these genes were transcription regulators, 13 % were involved in signal transduction, and 14 % were ROS-response genes. The primary enzyme that produces these radicals is the NOX family gene NADPH oxidase 2 (NOX2), which produces ${\text{O}}_2^{ \bullet - }$ [15, 16].

One natural dietary constituent (NDC) that may affect the ${\text{O}}_2^{ \bullet - }$ produced by NOX2 infiltrating proinflammatory cells is dark cocoa, which antagonizes NOX2 activation. [17] Cocoa contains relatively high amounts of the flavonoid epicatechin and has been found to have nearly twice the antioxidant content of red wine and up to three times that of green tea when assessed in vitro. One mechanism is that flavonoids can bind directly to some protein kinases and thereby antagonize the consequences of ROS-inactivated phosphatases (see below). Fish oil diet has also been shown to reduce inflammation in a rat colon carcinogenesis model. The suspected target is NOX2, but the mechanism has not been elucidated [18].

Regarding inflammatory cytokines, the NDC astaxanthin suppresses colon carcinogenesis in mice, partly through inhibition of the expression of inflammatory cytokines [19]. The combination of β-cryptoxanthin, a carotenoid, and hesperidin, a flavonoid extracted from the Mandarin orange, also suppresses the expression of proinflammatory cytokines [20], but the mechanism has not been described. Other NDCs, such as the antioxidant curcumin, a polyphenol extracted from turmeric, act through the inhibition of phosphorylation of the inhibitor of κB (IκB), which in turn reduces the nuclear translocation of nuclear factor-κB (NF-κB) and thereby silences expression of proinflammatory cytokines CXCL1 and CXCL2 [21, 22].

Other sources of RONS are the inflammatory cells themselves [4•, 14]. One is the recently discovered resident neutrophil. These unique cells are localized to the crypts and express the NOX family member NOX1 [23]. Presumably, these normal cells use this enzyme for defense against the fecal microbiota. On the negative side, when activated, these adherent lymphocytes are postulated to be involved in the pathogenesis of inflammatory bowel disease, and they may also be involved in initiating and maintaining the micro-inflammatory environment associated with sporadic colon cancer. Another are the infiltrating activated macrophages and monocytes that upregulate NOS2 activity in response to cytokines [13, 14].

Upregulated NOS2 underpins chronic inflammation in general and colon carcinogenesis in particular [24]. The STAT1 signaling cascade is one of the major pathways that upregulates NOS2 [25]. One antioxidant NDC that antagonizes NOS2 in colon inflammatory lesions and cells is the green tea constituent epigallocatechin gallate (EGCG). This compound inhibits activation of STAT1 by suppressing NOX-produced phosphorylation of the pro-STAT1 protein [26]. Another NDC is cyanidin and its metabolites. These antioxidant anthocyanidins are found in grape, blackberry, blueberry, cherry, cranberry, elderberry, and raspberry as well as apple, plum, red cabbage, and red onion. They work by attenuating NF-κB activity, which regulates NOX2 transcription through its binding site in the promoter of NOS2 [27]. Another antioxidant is the anthocyanidin resveratrol, which is obtained from grapes and wine. It inhibits NOS2 induction via inhibiting ROS activation of the p38 and ERK pathways. It also depresses ROS activation of NF-κB [28]. Another antioxidant is pterostilbene, which is found in grapes and blueberries. Its mechanism is as that for resveratrol [29, 30]. Eight other STAT-inhibiting NDCs, including capsaicin and curcumin, are discussed by Trecul and coauthors [31].

## ROS and NOS Production by Colon Cancer Cells

One way colon cells produce ROS is via xanthine oxidase (XO), which converts hypoxanthine to xanthine and then uric acid in the presence of molecular oxygen to yield ${\text{O}}_2^{ \bullet - }$ and H_2_O_2_. There is some evidence that the XO-derived ROS normally play a role in antimicrobial defense. 1′-acetoxychavicol acetate (ACA), an NDC present in members of the ginger plant family, is an inhibitor of XO enzymatic activity. It also upregulates Nrf2, which causes activation of phase II enzymes [32]. Another source of ROS is the mitochondria, wherein 1 % to 2 % of the O_2_ electrons used for producing adenosine triphosphate (ATP) leak from the electron transport chain as ${\text{O}}_2^{ \bullet - }$ and H_2_O_2_ [33].

Very important sources of ROS in the colon are the NOX enzyme complexes. Family members NOX1 and NOX4 produce abundant amounts of ${\text{O}}_2^{ \bullet - }$ and H_2_O_2_ in colon cells [6, 34]. They are expressed in the crypts and probably play a role in the innate response by the normal colon to fecal microbiota [35, 36]. They are unregulated threefold in precancerous lesions and in tumors. NOX-1 is activated in colon cells by the proinflammatory factor tumor necrosis factor (TNF)-α [37]. It is also upregulated by oncogenic ras [38, 39]. One of the earlier reports showing an important relationship between NOX1 and colon cancer cells was by Szanto et al. [40]. These authors showed that antisense oligonucleotides of NOX1 not only lowered ROS production but at the same time reduced the proliferation rate of the cells. Unfortunately, the literature is lacking information on any NDCs that can directly diminish the activity of NOX1 or NOX4. On the other hand, potentially all the antioxidant NDCs may be able to scavenge the ROS produced by these enzymes before they can produce damage to the cell.

NOX enzymes are protein complexes [41]. Activation of the oxidase activity is dependent on Rac1, a small G protein. The binding of growth factors (ie, TGF-α) to their receptors initiates signal transduction, which activates Ras. Ras transfers the signal to phosphatidylinositde-3 kinase (PI3K). Rac1 is a key downstream target of PI3K, and one of a number of the protein targets of PI3K kinase activity is a member of the NOX complex, NOXA1. Thus, Rac1 and NOXA1 facilitates NOX1 enzyme activity. This growth factor-ras-rac1-NOXA1 signaling pathway partially explains why colon tumor cells that harbor a mutant K-ras gene, or exhibit overexpression of growth factors and/or their receptors have constitutively activated NOX1 activity [38, 42–44].

The downstream targets of the PI3K–Akt pathway are often abnormally activated in colon because of gain-of-function mutations that lead to oncogenicity [45]. As a result, BCL2-antagonist of cell death (BAD) is inhibited and there is increased cell survival. Recent studies [46] show that flavonoids such as quercetin and epicatechin can bind directly to some protein kinases [47], including Akt/protein kinase, Janus kinase 1 (JAK1), mitogen-activated protein kinase kinase 1 (MEK1), PI3K, mitogen-activated protein (MAP) kinase kinase 4 (MKK4), and Raf1, and alter their phosphorylation state and their ability to regulate multiple cell-signaling pathways. The polyphenol antioxidant NDC ellagic acid (obtained from blackberries, cranberries, pecans, pomegranates, raspberries, strawberries, walnuts, wolfberry, grapes, and peaches) also prevents PI3K/Akt activation [48, 49].

The proinflammatory NF-κB, which is a commonly overactive gene in colon cancer, participates in the control of transcription of more than 3,000 target genes, including NOS2, cyclooxygenase 2 (COX-2, also called PTGS2), NOX1, NOX4, NOX2, interleukins, AKT1, TNF, EGFR, GADD45B, MAP2K6 (MEK6), NQO1, STAT3, TP53, BCL2A1, BCL-X, p21^CIP1/WAF1^, BIRC2, GADD45B, NQO1, PDGFB, PLAU, SOD2, REL, and MYC. Thus, NF-κB is involved in the inflammation, formation of DNA adducts, cell cycle regulation, apoptosis, angiogenesis, and invasion/migration/metastasis aspects of carcinogenesis [2••, 8, 33, 50, 51••]. Briefly, in the canonical pathway of activation, NF-κB is bound to IκBα. The IkappaB kinase (IKK) complex phosphorylates IκBα, thereby releasing it from NF-κB. NF-κB then translocates to the nucleus, and gene transcription ensues [51••]. There is also a second pathway for activating NF-κB that involves RONS. It has been shown that TNF-α and IL-1β activate NOX4 (which does not have NOXA1 or Rac1 within the complex) with a concomitant rise in ROS. The radicals then activate NF-κB activity [50]. The central player for this pathway is the redox switch protein LC8 [52]. LC8 interacts with IκBα in a redox-dependent manner. In cells exposed to TNF-α or H2O2, LC8 dissociates from IκBα. The released IκBα is then phosphorylated by the IKK complex. Thus, ROS can be responsible for activation of NF-κB. Interestingly, NF-κB and the other NADPH oxidase in colon cells (NOX1) regulate each other. This is brought about because the promoter of the NOX1 gene has a consequence sequence for NF-κB binding [41, 53]. Thus, there is a positive feedback loop in which NF-κB activation by NOX1-derived O2^●−^ and H_2_O_2_ leads to further induction of NOX1, with ever greater production of O2^●−^ and H_2_O_2_ and ever-increasing levels of damage to the cells. One of the means of cross-talk between infiltrating cells and epithelial cells is the STAT3-NF-κB axis of transcription [54•]. These genes are constitutively activated in colon cancer tumors. STAT3 regulates numerous genes and NF-κB targets more than 3,000. Activated infiltrating cells release proinflammatory cytokines that activate production of STAT3 and NF-κB in the epithelial cells. In turn, the epithelial cells exhibit increased proliferation, survival, and angiogenesis.

There are numerous antioxidant NDCs that affect NF-κB. Included within this group is lycopene (from tomatoes, carrots, red bell peppers, watermelons, and papayas) [55•]. It affects NF-κB through MAPK/ERK and PI3K/Akt signaling pathways that are plausibly dysregulated by RONS. Thus, lycopene inhibited cell proliferation of human colon cancer cells via suppression of the Akt signaling pathway and downstream targeted molecules. Other antioxidant NDC affecters of NF-κB are curcumin, the carotenoid astaxanthin, and sulforaphane [56]. Sulforaphane is obtained from cruciferous vegetables such as broccoli, brussel sprouts, cabbages, garlic, and onions. The anthocyanidin family, represented by delphinidin (from cranberries, Concord grapes, and pomegranates), also works by inhibiting NF-κB activation [57]. Pterostilbene, which is chemically related to resveratrol and is found in blueberries and grapes, reduced NF-κB activation by inhibiting the phosphorylation of PKC, and decreased downstream target gene expression, including NOS2, COX-2, and nuclear factor (erythroid-derived 2)-like 2 (Nrf2) signaling [30].

Proinflammatory cytokines upregulate NOS2 in colon epithelial cells [4•]. NOS2 has a major role in the activation of COX-2, which is an immediate—early response proinflammatory gene that is upregulated in nearly all colon cancers [58]. COX-2 indirectly regulates gene expression through metabolizing arachidonic acid (AA) to prostaglandin E_2_ (PGE_2_). This eicosanoid binds and activates EP receptors. The receptors regulate several signal transduction pathways (eg, PKC to ERK, and epidermal growth factor receptor [EGFR] to AKT). These pathways affect cell proliferation, invasion, survival, and angiogenesis [2••, 59]. Interestingly, in liver cells, NOX1 activation has been shown to upregulate COX-2 expression and activity, which in turn induced NOX4 expression and ROS production. Taken together, these results indicate that the NOX proteins in colon and COX-2 are reciprocally regulated [60, 61].

Liberation of arachidonic acid by cytosolic phospholipase A(2) (cPLA[2]) is often the initial and rate-limiting step in leukotriene (LT) and PG biosynthesis. Because COX-2 enhances the formation of a cPLA(2)alpha-iNOS binding complex, it appears that COX-2—induced augmentation of cPLA(2)alpha S-nitrosylation is mediated at least in part through increased association between NOS2 and cPLA(2)alpha [62]. Therefore, therapy aimed at disrupting this interplay may represent a promising strategy to effectively inhibit PGE_2_ production and thereby prevent inflammation and carcinogenesis.

Numerous antioxidant NDCs have been identified that inhibit COX-2. Some of these work indirectly through inhibiting NF-κB. Included in this list are curcumin, olive oil, resveratrol, EGCG, the flavone apigenin (from leafy vegetables [eg, parsley, artichoke, basil, celery]), the isoflavone genistein (from fava beans and soy beans), the flavonoid kaempferol (from tea, broccoli, grapefruit, and apple), the flavonoid chrysin (from honey), and sulforaphane [9, 63]. Some of these may actually be affecting NF-κB expression in the activated macrophages of the inflammatory lesion.

Arachidonic acid is also metabolized by 5-lipoyxgenase (5-LOX) to LTs. The enzyme and its metabolites are elevated in adenomas and are even higher in cancer tumors. LTs are potent inflammatory mediators that act by stimulating the G protein—coupled receptors BLT1 and BLT2 that in turn induce NF-κB, TNF-α, and ILs in the activated macrophages and leukocytes within the inflammatory lesion. Some LOX products are procarcinogenic, while others are anticarcinogenic. The procarcinogenic enzyme 5-LOX leads to the production of LTs. 15-LOX-1 has anticarcinogenic effects. In human colon epithelium, 15-LOX-1 expression levels are low and NSAIDs induce its expression. Red ginger has the potential to decrease eicosanoid levels, perhaps by inhibiting their synthesis from arachidonic acid. Alternatively, this antioxidant may work indirectly through scavenging of NO^●−^ [64].

## RONS and Mutagenesis

The sequence of some of the genetic changes that correlate with the observed pathological transformations that occur during the initiation and promotion/progression stages of colon carcinogenesis was identified more than 25 years ago [1, 2••, 65]. It begins with loss of APC function, as the tissue changes from normal appearing into an early adenomatous lesion. Aneuploidy and methylation events arise in the early adenomatous lesion, and microsatellite instability, K-ras mutations, and upregulation of COX-2 develop as the early adenoma evolves into an intermediate adenoma. As this lesion progresses to the late adenoma stage, there is loss of heterozygosity of the genes deleted in colorectal carcinoma (DCC) and SMAD family member 4 (SMAD4). Lastly, just before the onset of clinical cancer, the p53 gene is mutated. These genetic aberrations are associated with the formation of ROS- and NOS-caused 8-hydroxy-deoxyguanosine (8-OH-dG) adducts in the DNA [67]. The result is a GC➔TA base pair transversion and/or an apurinic/apyrimidinic site if not repaired before DNA replication occurs. Thus, 8-OH deoxyguanosine bases frequently result in mutations in genes (eg, K-ras and p53). In addition, RONS cause single- and double-strand breaks, with the result of loss and gain of genetic information. Radicals also cause DNA-protein cross-links and pyrimidine and purine lesions. These adversely affect the integrity of the genome, with genomic instability as one consequence. Another effect of RONS is reduction of 5-methyl-deoxycytosine content in DNA [66, 67]. The presence of oxidized deoxyguanosine reduces ability of the DNA to serve as a substrate for DNA methyltransferase (DNMT). Hence, the enzyme does not transfer the methyl group from methionine to deoxycytosines during DNA replication, and the level of 5-methyl-cytosines becomes progressively reduced [67]. This hypomethylation of the cell’s genome correlates with the onset of genomic instability, microsatellite instability, and increased mutation rate. DNA hypomethylation also causes activation of genes normally transcriptionally downregulated by hypermethylation [68] of their promoter region (ie, the oncogenes H-ras and c-MYC, which are frequently overexpressed by colon cancer cells). Excess RONS also leads to shutting off tumor suppressor genes by hypermethylation of their promoter. However, the mechanism producing this phenomenon remains obscure.

Antioxidant NDCs scavenge hydroxyl radicals and other ROS. Apple polyphenols have been shown to elevate expression of the phase II genes [69]. Tea polyphenols also induce phase II enzyme activity. Another constituent is the antioxidant dithiolethione from cruciferous vegetables. It activates Nrf2 (see below) signaling, which in turn induces phase II enzymes. Others are sulforaphane, indole-3-carbinol, and phenethyl isothiocyanate (PEITC) (from cruciferous vegetables) [70]. The coffee constituents 5-O-caffeoylquinic acid and N-methylpyridinium have also been identified as inducers of the Nrf2-regulated detoxifying pathways [71].

Antioxidant NDCs also target the epigenome. Among these are the polyphenols from green tea, apples and coffee, genistein, soy isoflavones, curcumin, ellagitannin, indole-3-carbinol, lycopene, sulforaphane, PEITC, and phenylhexyl isothiocyanate (PHITC) [72]. Also effective is resveratrol, which affects the activity of histone deacetylase SIRT1, which regulates the activity of DNMT1 [73]. Others are inhibitors of histone acetyl transferases (anacardic acid from cashew nut), garcinol (from mangosteen), and ursodeoxycholic acid (a metabolic byproduct of intestinal bacteria). Finally, the relatively unexplored modulators of histone lysine methylation (chaetocin, a fungal metabolite, and n-3 polyunsaturated fatty acids) may be effective in inhibiting changes in the epigenome [74]. Also effective in targeting the epigenome are black raspberries (BRBs). We recently demonstrated that daily oral administration of BRBs for 4 weeks resulted in protective modulation of multiple epigenetic biomarkers (SFRP2, SFRP5, PAX6a, and WIF1) of the WNT pathway that was modulated in a protective direction by BRBs in healthy tissues and in colorectal tumors in cancer patients. This observation was associated with decreased expression of DNMT1 [75].

## RONS-Regulated Signaling Pathways

Low levels of RONS serve as second messengers to regulate multiple signaling pathways as a way to maintain cellular homeostasis [76]. RONS accomplish this by using switching proteins that are reversibly oxidized by H_2_O_2_ and returned to their normal state by reducing enzymes. One switch position is brought about through cysteines (Cys) on the surface of these proteins. The thiol moiety (-SH) of these Cys is highly, and in some proteins specifically, sensitive to oxidation, with the results of the formation of a sulfenyl moiety that can form a disulfide bond with another oxidized surface Cys. Consequently, the protein acquires a structural change and is either inactivated or indirectly activated (see ASK1 below). The switch is flipped back by reduction of the Cys by thioredoxin (Trx), thereby establishing “on” and “off” states of molecular pathways regulated by RONS [76–79].

With respect to carcinogenesis, foremost among the ROS inactivation switch proteins are members of the large family of PTPs [76–79]. For example, PTPs have an essential role in downregulating receptor tyrosine kinase (RTK)-mediated signaling by dephosphorylating RTKs. On the other hand, PTP inactivation by NOX-derived ROS enhances RTK tyrosine phosphorylation which leads to amplified activity of the ERK pathway, suggesting that modulation of RTK signaling via PTP inactivation by NOX-derived ROS may have a significant contribution in mediating the activation of ERK/MAPK pathway. Another example is the PTEN. Normally, PTEN dephosphorylates phosphoinositide substrates, thereby negatively regulating the intracellular levels of phosphatidylinositol-3,4,5-trisphosphate. The consequence is negative regulation of the Akt signaling pathway [80]. Alternatively, inactivation of PTEN constitutively activates the AKT pathway, a major player in colon cancer carcinogenesis. Most loss of PTEN is attributed to deletions and hypermethylation [65]. However, ROS may also be a player, as when Cys on PTEN are oxidized, the enzyme is inactive, and the Akt pathway is upregulated and cell survival is augmented.

Another ROS-targeted phosphatase activity is the gene dual specificity protein phosphatase (DUSP) [76–79]. DUSP negatively regulates the activity of c-Jun N-terminal kinase (JNK) and p38 mitogen-activated protein kinase (p38) stress signaling pathways in a way analogous to PTEN. When DUSP protein is oxidized, its phosphatase activity is inactivated and stress signaling pathways are upregulated. Thus, by oxidizing DUSP and activating JNK and P38, several important cellular functions, including cell growth, differentiation, survival, and apoptosis, are dysregulated. The Cdc25 phosphatases (A, B, and C) that participate in cell cycling are also oxidized and inactivated by ROS. Central to the cell-cycling process are the CDKs and their catalytic partners, the cyclins. The CDKs are negatively regulated by CDK inhibitors (eg, p16, p21, and p27) and positively activated by CDK-activating kinases. CDC25 regulates the extent of phosphorylation of CDKs at differing times of the cell cycle. Accordingly, when CDC25 is oxidized and inactive, the CDKs are fully activated throughout the cycle and the cells proliferate faster. Meeran and Katiyar [81] have discussed the NDCs that affect the cell-cycling pathway.

The apoptosis signal-regulating kinase 1 (ASK1), which is a member of the MAPK family, is indirectly activated by RONS [76–79]. ASK1 elicits numerous cellular responses to stress through its activation of the JNK and p38 pathways. As noted above, Trx is an antioxidant enzyme. Under normal conditions, it binds to ASK1 and inhibits its kinase activity. Under oxidative stress conditions, the two cysteine residues in the active site of Trx undergo reversible oxidation to form a disulfide bond with each other. Thus, the conformation of Trx protein is changed, ASK1 dissociates from Trx, and its kinase activity is turned on.

Another transcription factor activated by RONS is Nrf2 [76–79]. Nrf2 is retained in the cytoplasm and destroyed in the proteasome when bound to Keap1. Reactive species oxidize Keap1, thereby permitting Nrf2 to translocate into the nucleus and activate a myriad of oxidative response genes, whose resultant enzymes extinguish the negative effects of the elevated levels of RONS. Many antioxidant NDCs increase Nrf2 activity. Interestingly, sulforaphane amplifies Nrf2 activity by chemically modifying cysteine thiols of Keap1 [82]. Cinnamon is also an activator of Nrf2 [83].

The WNT canonical pathway is commonly activated in colon cancer [65]. NOX1 and NOX4 have been implicated in this inappropriate upregulation [6]. In this pathway, the WNT factor causes the translation of the transcription factor β-catenin (β-Ctn) into the nucleus. The primary means of activation of the WNT pathway in colon cancer is through deletions in the adenomatous polyposis coli (APC) gene. Mutations of APC are considered to be early events in colon carcinogenesis, thereby explaining why inappropriate expression of the WNT pathway is one of the earliest events leading to colon cancer [65]. In normal cells, APC forms a complex consisting of β-Ctn, casein kinase-1-α, glycogen synthase kinase-3 (GSK3), and axis inhibitor. GSK3 phosphorylates β-Ctn, thereby priming it for degradation by the proteasomes. When the WNT factor binds and activates its receptor frizzled, the signal is passed to disheveled (Dvl). Dvl binds with the β-Ctn-APC complex and inhibits GSK3 activity. β-Ctn then translocates into the nucleus to activate its target genes. Among these are CD44, c-Myc, c-Jun, Fos-related antigen-1, cyclin-D1, transcription factor-1, and COX-2. If APC is inactivated, phosphorylation of β-Ctn does not occur and it translocates into the nucleus, even in the absence of WNT [84].

Reactive species initiate a second way to activate translocation of β-Ctn into the nucleus [85]. This is brought about by ROS oxidizing the nucleoredoxin (Nrx) protein [86]. In its reduced state, Nrx binds to Dvl. When Nrx is oxidized, it releases Dvl to bind with the β-Ctn-APC complex. With Dvl in the complex, the kinase activity of GSK3 is blocked and β-Ctn translocates into the nucleus to activate specific target genes. The source of ROS that oxidizes Nrx in the colon has been shown to be NOX1. Another frequently dysregulated signal pathway in colon cancer is Notch. It has been speculated that Notch activation might be an essential event triggering colorectal cancer. Although the molecular mechanism of notch activation is incompletely understood, a strong role for NOX1 has been implicated. Thus, again, NOX1-produced ROS plays a seminal role in colon carcinogenesis.

Quercetin, an NDC available from black and green tea, capers, apples, onions, red grapes, citrus fruit, tomato, broccoli, and a number of berries (including raspberry, cranberry, chokeberry, buckthorn, and the fruit of the prickly pear cactus) is a strong disruptor of dysregulation of the WNT pathway. Others are EGCG, curcumin, sulforaphane, soy isoflavone, epigallocatechin-3-gallate, resveratrol, lycopene, and piperine (from black pepper) [55•, 87, 88].

## Micrornas, RONS, and Inflammation

MicroRNAs (miRNAs) are short, noncoding RNAs that regulate cell homeostasis by inhibiting translation or causing degradation of the mRNA of target genes. When inappropriately expressed, they can affect the expression of tumor suppressor genes and oncogenes. In addition, through their actions on mRNAs, they can cause epigenetic modifications of the DNA, genomic amplifications and deletions, cellular stress, and inflammation [89–91]. It has recently been reported that some miRNA expression is governed by the binding of the proinflammatory NF-κB transcription factor to the consequence sequence of the miRNA promoter region [93]. Inflammation also elicits changes in expression of miRNA, primarily through the actions of proinflammatory cytokines. Specifically, proinflammatory signaling cytokines IL-1β, IL-6, and TNF-α alter the expression profile of miRNAs and the promoter region of miR-146a, which has a consequence sequence region for NF-κB [92]. Furthermore, the expression profile of miRNAs can be altered by exogenous H_2_O_2_ [93]. Thus, inflammation and ROS are disruptors of normal miRNA expression. Colon cancer cells exhibit numerous changes in their miRNA profile as compared with that of the normal cells/tissues that dysregulate expression of numerous genes that are involved in all aspects of colon carcinogenesis.

Several NDCs affect miRNA profiles. Included in this collection are EGCG, apple polyphenols, coffee polyphenols, ellagic acid, genistein, parthenolide (from chrysanthemum) curcumin, ellagitannin, and indol-3-carbinol. A major target of miRNAs is regulation of NF-κB. Excellent reviews discussing NDCs and miRNAs relationships were recently published by Li et al. [94••] and Parasamka et al. [95•].

## Conclusions

**Fig. 1:**
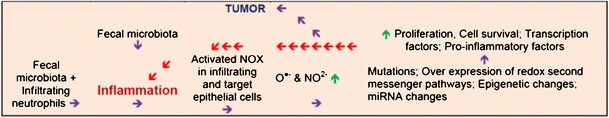
A steady-state of elevated RONS and pro-inflammatory factors is a formative driver of colon carcinogenesis. The thesis of this review is that colon cancer chemopreventative foods and their active constituents work through ameliorating the molecular pathways that are dysregulated by a continous cycle of pro-inflammatory factors and free radicals (depicted by the *red arrows*). The initiating cause of inflammations unclear, but the fecal microbiota is one candidate. Oxidative stress activates a variety of transcription factors including NF-κB, p53, β-Ctn-Wnt, and Nrf2 which results in the expression of over 5,000 different genes, including those for growth factors, inflammatory factors, and cell cycle regulatory molecules. The paramount feature of the vast majority of chemopreventive substances is their ability to antagonize pro-inflammatory factors and reduce the level of RONS. Therefore, it is expected that molecular pathways most commonly affect by chemopreventive agents would be those that are dysregulated by pro-inflammatory factors and free radicals. *Green arrow* signifies up-regulation and *blue arrows* signify progression of processes

A steady state of elevated RONS and proinflammatory factors is a formative driver of colon carcinogenesis (Fig. 1). The intention of this review is to assess the thesis that colon cancer chemopreventive foods and their active constituents work through ameliorating the molecular pathways that are dysregulated by proinflammatory factors and free radicals. Extensive research has revealed that continued oxidative stress leads to chronic inflammation, which in turn mediates most chronic diseases, including colon cancer. Oxidative stress activates a variety of transcription factors, including NF-κB, AP-1, p53, β-Ctn-Wnt, and Nrf2, which results in the expression of more than 5,000 different genes, including those for growth factors, inflammatory cytokines, chemokines, and cell cycle regulatory molecules [24, 96]. The information in this review clearly implicates the role of RONS in different phases of inflammation and carcinogenesis. Therefore, targeting redox-sensitive pathways and transcription factors with antioxidative NDCs offers great promise for cancer prevention and therapy. We have outlined numerous agents that can interfere with redox-regulated cell signaling pathways. However, NPCs that antagonize the enzymes responsible for much of the endogenous production of ROS (ie, the NOX family members 1, 2, and 4) are rare. One exception is mastic resin produced from the evergreen shrub *Pistacia lentiscus*. It has been shown to decrease inflammation in Crohn’s disease patients [97]. In addition, Triantafyllou et al. [98] reported that whereas TNF-α significantly increased the cellular superoxide production by NOX1 and 2, mastic gum completely abolished this stimulation. Furthermore, mastic gum inhibited the activity of PKC activity in cell homogenate, suggesting that NDC inhibited phosphorylation of NOXA1 by PKC. Thus, mastic resin may antagonize activation of these two NADPH oxidases. On the other hand, the increase of NOX4 in colon epithelial cells would not be affected. We hope continued research will soon identify several and they may be used to reduce the incidence of colon cancer.
